# Does Warm-up Type Matter? A Comparison between Traditional and Functional Inertial Warm-up in Young Soccer Players

**DOI:** 10.3390/jfmk5040084

**Published:** 2020-11-19

**Authors:** Giovanni Fiorilli, Federico Quinzi, Andrea Buonsenso, Giulia Di Martino, Marco Centorbi, Arrigo Giombini, Giuseppe Calcagno, Alessandra di Cagno

**Affiliations:** 1 Department of Medicine and Health Sciences, University of Molise, v. De Sanctis 1, 86100 Campobasso, Italy; fiorilli@unimol.it (G.F.); giuseppe.calcagno@unimol.it (G.C.); 2 Department of Motor, Human and Health Sciences, University of Rome “Foro Italico”, Lauro de Bosis Square, 15, 00197 Rome, Italy; fquinzi@libero.it (F.Q.); giulia.dimartino21@gmail.com (G.D.M.); marco.centorbi@hotmail.it (M.C.); arrigo.giombini@uniroma4.it (A.G.); alessandra.dicagno@uniroma4.it (A.d.C.)

**Keywords:** physical activity, exercises, muscle strength and endurance, sport medicine, joint stability

## Abstract

Functional inertial training, a popular high-intensity training mode, provides high neuromuscular activation, developing proprioception, postural control, power, and sprint time. Aim of the study was to assess the acute effects of two types of warm-up (WU), inertial warm-up (IWU) vs. traditional warm-up (TWU), on explosive and reactive strength, sprint, and Change of Directions (COD) in young soccer players. In a randomized cross-over design study, twelve soccer players (aged 13.3 ± 0.7) performed 16 min of IWU and 16 min of TWU. IWU and TWU were spaced two weeks apart. Pre and post intervention tests, aimed at assessing explosive and reactive strength, sprint, and COD ability included: Squat Jump test (SJ), Countermovement Jump test (CMJ), Drop Jump test (DJ), Seven Repetition Hopping test (7R-HOP), 40 m-sprint test (40 m), and Illinois Agility Test (IAT). RM-ANOVA, used to compare differences between IWU and TWU effects (the level of significance set at *ρ* ≤ 0.05), showed enhanced performance after the IWU compared to the TWU. In addition, the effects of the IWU on performance lasted longer after the IWU than after the TWU. For IAT, the enhanced effects of IWU on performance lasted up to ten minutes after the administration of the IWU. Our results suggest that IWU affects functional changes displaying earlier adaptation in explosive and reactive strength with longer lasting effects compared to TWU and it could be recommended in young soccer athletes as a WU procedure.

## 1. Introduction

Warm Up (WU) is considered essential to enhance the subsequent performance and optimally prepare the soccer players for jumps, sprint, change of directions (COD), and agility tasks. Change of direction and agility tasks require a complex synchronization of different movements and multiple strength components [1]. In particular, eccentric strength plays a crucial role in COD, which requires the body to decelerate and stabilize as quick as possible and to re-accelerate in a new direction, and in jumping action during the braking phase [2]. The performance in COD and agility tasks has been shown to be correlated with a combination of maximum and reactive strength and power [3]. Moreover, eccentric hamstring strength was the primary predictor of agility deceleration task [4]. Furthermore, agility is a multifaceted quality, which recognizes, in addition to the physical demands, cognitive processes and technical skills too [2].

Traditionally, soccer WU protocols for young athletes consist of dynamic movements which aim to increase core body temperature [5], to enhance motor unit excitability, and active ranges of motion [3]. In addition, WU dynamic exercises create an optimal environment for power production by enhancing neuromuscular function [5].

Recently, different WU modalities have been proposed as an alternative to traditional WU (TWU). To this respect, flywheel devices, providing an accentuated eccentric load applied to WU exercises, may enhance WU efficacy. These devices use the principle of the kinetic energy accumulation in a flywheel, resulting in an accentuated eccentric phase that may ultimately enhance high neuromuscular activation, postural control, muscle coordination, and power and sprint time [6,7]. Thanks to its adjustable resistance and individualized eccentric overload [8], exercises with the flywheel application could be included in young soccer athletes’ WU. The training load of this technology is mainly regulated by increasing the speed of movement [9], providing a resistance in the eccentric phase proportional to the one generated by the athlete’s concentric effort. Moreover, the flywheel device provides in the eccentric phase different loads in each repetition. The absence of athlete knowledge of the variable load information stimulates different and constant adaptations of this neuromuscular system in any exercise repetition [10]. Since soccer is a sport in which the uncertainty, both of the loads and the work environment, characterizes performance, young athletes could benefit by adhering this protocol, which applies similar stressors [11].

It is well known that the energy needed to perform eccentric actions is about one fifth of the one required for concentric action of the same cycle [12]. This is crucial to the efficacy of a WU protocol in which it is mandatory not to fatigue an athlete [13].

Several studies showed that functional inertial training, performed at least once weekly, improves task specific performance in soccer [11,14].

To the best of our knowledge, no previous study has investigated the acute effects of an inertial eccentric load WU on young soccer players. Kale and colleagues [15] studied the acute effects of flywheel application on adult athlete performance with no significant results. A thorough quantification of the effects of inertial WU (IWU), as compared to a TWU, would allow coaches and athletes to choose which method best suits for their sport.

The aim of the present study was to assess the acute effects of a flywheel device, employed in a WU, on explosive and reactive strength, sprint, and COD, comparing these results with those of a TWU. It was hypothesized that young soccer players would benefit by adhering to a specific WU protocol based on accentuated eccentric loads, used in similar conditions in which they compete.

## 2. Materials and Methods

### 2.1. Study Design

The present study is a randomized crossover trial in which young soccer players receive two treatments in a random order, designed to evaluate the acute effects of two WU protocols, IWU vs. TWU, on explosive and reactive strength, linear sprint, and COD performance and to assess accentuated eccentric load benefits, applying a cone-shaped flywheel device in sport specific conditions.

### 2.2. Participants

Twelve male young soccer players (aged 13.3 ± 0.7; weight 56.81 ± 4.31 kg; height 1.66 ± 0.05 m; BMI 20.62 ± 1.34 kg/m^2^) volunteered to participate in the present study. All participants had at least 4 years of experience and usual training practice included 3 field-based sessions, lasting approximately 120 min. The athletes had no previous experience with the use of any type of flywheel device. They were familiarized to accentuated eccentric training, performing 4 sprint trials with the device on their back. The sample size (12 total participants) was calculated *a priori* using G*Power 3.1.9.7 (G*Power software, Dusseldorf, Germany). This sample size was calculated in order to have an α error probability = 0.05 and a Power = 0.80 with Pillai’ V = 0. After being informed about the study procedures, risks, and aims, participants’ parents signed the written consent. The study was designed and conducted in accordance with the Declaration of Helsinki and approved by the bioethical local committee of University of Rome “Foro Italico” (University Committee for Research (CAR-IRB), Code: CAR49/2020, 26 May 2020).

### 2.3. Procedures

After the familiarization session at baseline, all participants underwent a testing session in order to assess explosive and reactive strength levels, linear sprint velocity, and COD performance.

Lower limb explosive and reactive strength were assessed via Optojump (Microgate, Bolzano, Italy). It has an excellent test-retest reliability ranging from 0.982 to 0.989 [16]. Explosive strength was assessed by means of the Squat Jump (SJ), Countermovement Jump (CMJ), and Drop Jump (DJ) whereas the Seven Repetition Hopping (7R-HOP) test was used to measure the reactive strength. Following three attempts, interspersed by 45 s, the highest flight time (ft) of SJ (SJ_ft), CMJ (CMJ_ft), and DJ (DJ_ft) were chosen for analysis.

In the SJ test, participants were required to assume a static squat position with knees flexed at 90°, performing a concentric action with the instruction to jump as high as possible, with free arms.

In the CMJ test, participants were required to jump as high as possible, keeping hands on their hips. They started with a preliminary countermovement, reaching a bending of 90°.

In DJ, the athletes stepped down from a measured drop height (0.5 m), landed on the ground and performed a maximal effort vertical jump. Participants were instructed to maximize jump height and minimize ground contact time (ct).

In the 7R-HOP Test, participants were asked to perform seven continuous jumps with free arms, with the smallest possible countermovement amplitude and a shortest ground ct. Jump_ft was assessed for each jump, and the average Jump_ft was computed across the seven jumps (7R-HOP-ft). Similarly, for each jump, ct was computed and averaged across jumps7R-HOP-ct. The test reliability between and within sessions is 0.40–0.90 and 0.87–0.98, respectively [17].

The Illinois Agility Test (IAT) test was used to evaluate the COD performances. The test reliability is 0.8–0.9 [18]. Field structure details are provided. The athlete, on command, had to sprint 9.2 m and to return to the starting line; then he had to swerve in and out of the markers; finally, he had to sprint another 9.3 m and to complete the test by passing through the finish gate. The time spent between the start and finish gate photocell was recorded by software (Microgate, Bolzano, Italy).

To assess sprint velocity, athletes were evaluated over a 40 m linear meter sprint test (40 m) with photelectric cells (Racetime2, Microgate, Bolzano, Italy). This test showed good to excellent reliability equal to 0.79–0.94 [19].

### 2.4. Experimental Setup

In this study, we used “The Flyconpower conical machine” (Cuneo; Italy) to administer the IWU. Participants were attached to the flywheel device by means of a rope positioned at their waist. This condition allows the athlete to move freely in multiple directions of the space [20].

### 2.5. Intervention

Participants were randomly divided into two groups: Group A and Group B, each comprising six participants. The randomization process was performed by assigning a progressive number to each participant. Successively, an online software (https://www.random.org/sequences/, Dublin, Ireland) was used to generate a random number list (from 1 to 12 with no repeated numbers). Based on this random number list, participants were assigned to Group A and B alternately.

Group A performed TWU first, and after two weeks, IWU. Group B did the opposite starting with the experimental IWU first, and TWU two-weeks later. Both groups performed WU, in either modalities, at the same time of the day. The two-week wash-out period between the two interventions was planned to reduce possible carry-over effects. Both protocols consisted of 16 min WU exercises. At the end of each WU, participants were evaluated, with the same battery of test used at baseline. In both WU protocols, the post tests were performed immediately after the WU (T1), after 5 min (T5) and after 10 min (T10). In the post WU assessments, only one attempt for each test was performed, to ensure the correct timing of the procedures.

TWU and IWU consisted of 16 min exercises. TWU performed their usual warm-up consisting of a combination of running exercises (4–5 min at light intensity), alternated to brief sprint periods, six minutes of plyometric exercises (high knee skips, repeated bouts of consecutive DJs from a 0.5 m high box, and speed ladder drills), and five minutes of dynamic mobility exercises for hips, arms, and trunk alternated to technical exercises with the soccer ball. 

**Figure 1:**
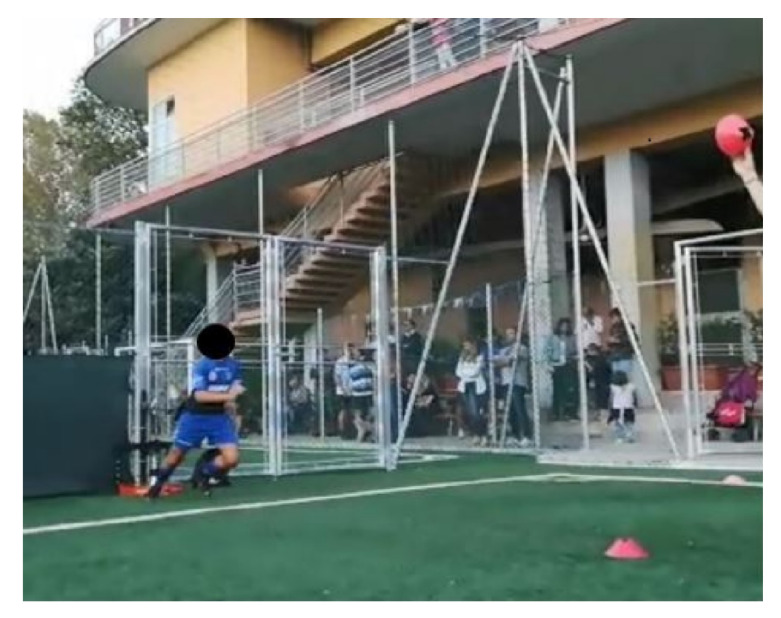
The isoinertial device application on the athlete exercises in the intervention phase.

IWU was the same of TWU, except for six minutes of exercises with flywheel device application, instead of plyometric exercises. Participants completed two familiarization sessions before IWU. The IWU protocol consisted of 4 m linear and diagonal sprints with the flywheel device fixed on the participant waist (4 sets × 10 repetition, 60 s of rest between sets), keeping the back to the device. Each participant was asked to sprint as fast as possible to the left or to the right towards a target gate, which was randomly indicated by the coach and successively, they returned in the start position, resisting the force generated by the flywheel device, encouraged to apply the maximum effort during the concentric phase, delaying the breaking action until the end of the eccentric phase (Figure 1).

### 2.6. Statistical Analysis

All the statistical tests have been carried out using the Statistica software (StatSoft, v10). All variables were tested for normal distribution using the Shapiro-Wilk test. For each variable (40 m; IAT; SJ_ft; CMJ_ft; DJ_ft; DJ_ct; 7R-HOP_ct; 7R-HOP_ft), a 2 × 4 repeated measure analysis of variance (RM-ANOVA) was employed to test the effect of WU (TWU vs. IWU) and time of measurement (hereinafter time; T0; T1; T5; T10). The Tukey honestly significant difference was used as a post hoc test. For all statistical tests, α was set at 0.05. Achieved power (1–β) and effect size (µ_p_^2^) were reported.

## 3. Results

The RM-ANOVA revealed a significant time by WU interaction on 40 m. The post hoc test showed that after TWU, sprint time at T0 was significantly longer than at T1 (*p* = 0.002) and at T10 (*p* = 0.001). In IWU, 40 m time at T0 was significantly longer than at T1 (*p* < 0.001).

For IAT, a significant time by WU interaction was observed. IAT time at T10 was shorter in IWU than in TWU (*p* < 0.001).

Significant effects of WU, time, and WU by time, were observed for SJ_ft. The post hoc showed that SJ_ft was longer in IWU (0.49 s) compared to TWU (0.45 s; *p* < 0.005). In TWU, SJ_ft at T0 was comparable to those observed at T1 T5 and T10 (all ps > 0.05); in IWU protocol, SJ_ft at T0 was shorter than at T1, T5, T10 (all ps < 0.05). SJ_ft at T10 was longer in IWU than in TWU.

A significant effect of WU, time, and WU by time interaction were observed for CMJ_ft. The post hoc test showed that CMJ_ft was longer in IWU (0.52 s) compared to the TWU (0.48 s; *p* = 0.001). In TWU, CMJ_ft at T0 was comparable to those observed at T1, T5, and T10 (all ps > 0.05), in IWU, ft at T0 was shorter than at T1, T5, and T10 (all ps < 0.001). CMJ_ft at T5 was longer in IWU than in TWU.

A significant effect of time and WU by time interaction were observed for DJ_ft. The post hoc analysis showed that DJ_ft lasted more in IWU (0.49 s) compared to TWU (0.47 s; *p* = 0.012). In both WU, DJ_ft at T0 was shorter than at T1, T5, and T10 (all ps < 0.001). In IWU, DJ_ft at T1 (*p* = 0.012) and T5 (*p* = 0.003) lasted longer than in TWU.

Significant effect of WU, time, and WU by time interaction was observed for 7R-HOP_ft. The post hoc analysis showed that 7R-HOP_ft lasted longer in IWU (0.46 s) compared to TWU (0.44 s; *p* < 0.001). At T5 and at T10 after TWU, 7R-HOP_ft was reduced compared to IWU (*p* = 0.019 and *p* < 0.001, respectively).

For DJ_ct, a significant effect of WU and WU by time interaction were observed. The post hoc showed that in IWU, DJ_ct (0.65 s) was longer than in TWU (0.60 s; *p* < 0.001) and that DJ_ct at T1 and at T5 was significantly shorter in TWU compared to IWU (*p* = 0.004; *p* = 0.019, respectively).

**Figure 2:**
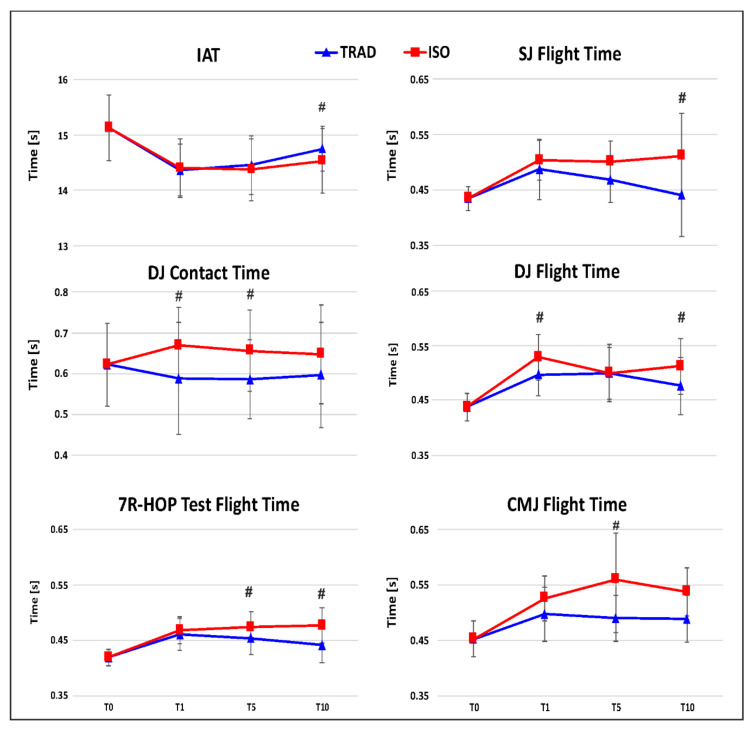
Time of Illinois Agility Test (IAT), flight time for the Squat Jump (SJ), Countermovement Jump (CMJ), and Seven Repetition Hopping Test (7R-HOP); flight time and contact time for the Drop Jump (DJ) test at the intervals considered in the present study (T0; T1; T5; T10). # denotes significant difference between the two protocols.

7R-HOP_ct did not differ between TWU and IWU. No effect of time and no interaction were observed for 7R-HOP_ct (Table 1; Figure 2).

## 4. Discussion

The efficacy of inertial training method in soccer has been demonstrated by the results of several studies [7,9,11]. Nevertheless, few studies have investigated the acute effects of the flywheel resistance application [8,15]. Kale and colleagues [15] used a flywheel device on 4RM half squat workouts in adult athletes, with no significant differences between pre and post sprint and vertical jump. Tesch [8] highlighted the superior acute efficacy of exercises with a flywheel device in augmented contractile force due to a larger muscle involvement than other resistance methods.

The purpose of the present study, was to investigate the IWU acute effects on explosive and reactive strength and compare IWU with a TWU protocol in young soccer players, using the flywheel device application directly on the task-specific soccer movements. Results showed that explosive and reactive strength, as assessed by SJ, CMJ, and DJ tests, were enhanced after IWU more than after TWU, and this enhancement after IWU lasted longer compared to TWU.

Several studies demonstrated that the exercise protocols in which the movement eccentric phase is accentuated, as in flywheel application, warrant greater strength improvements than those in which the load is constant during both the concentric and eccentric phases [9,20]. The prolonged eccentric exposure enhanced the strength performance [21]. Tous-Fajardo et al. [22] measured the surface electromyography activity in soccer player during the application of a flywheel device. They reported global electromyographic activity during a single bout of eccentric exercises, which facilitated major neuro-muscular adjustments than other strength methods.

It was hypothesized that the longer-lasting effects on the explosive and reactive strength were due to the intensity and duration of the muscular tension resulting in longer contraction periods, according to the stimulus-tension theory [23]. During the exercises with a flywheel device application, the continuous cyclic nature of repetition, consisting of the concentric phase immediately followed by the prolonged eccentric one with gradual controlled lowering, involved high levels of activation and force throughout each set [24]. The flywheel application produced concomitant enhancement for both concentric and eccentric phases for a longer time under tension (TUT) condition [25].

Considering that early adaptations to resistance performance are explained by neural adaptations, IWU could have induced a mechanism responsible for Post Activation Potentiation (PAP) [26]. Cuenca-Fernandez et al. [27] showed that flywheel intervention enhanced neuro-motor performance more than other types of weight exercises, with a significant impact on speed performance. These authors attributed this outcome to a PAP effect.

DJ_ft improved after both the WU protocols. TWU, including plyometric exercises, also may have induced good levels of PAP during DJ, eliciting the recruitment of motor units [26].

After IWU, DJ_ct was longer than after TWU. Differences of DJ_ct might be targeted for different performance goals. In soccer performance, players need longer times of take off when performing headshot skills, whereas shorter contact times are required in COD and other situations in which acceleration is the most important factor. Based on these considerations, both plyometric and IWU, that stimulate shorter and longer time of take-off, respectively, are advisable for soccer WU.

The 7R-HOP_ft was significantly increased after IWU than TWU, especially at T5 and T10. This test assessed tendon stiffness, which plays a critical role in rapidly transmitting strength from the muscles to the skeletal system. Stiffness allows a greater speed recoil of the muscle-tendon units, positively influencing the transition (eccentric-concentric) point of the stretch shortening cycle (SSC), increasing stretch reflex activity [28]. Flywheel application ensures the stabilizer muscle activation, by means of agonist and antagonist activation, which contribute to changes in stiffness during the 7R-HOP and to prevent injuries [29].

Athletes reached significant improvements in performing the IAT after both the WU. During IWU intervention, the flywheel device was applied directly to the participants’ waists [11]. A task-specific neural adaptation, due to the COD repetition, may have guaranteed the significant improvements. Nevertheless, performance time at T10 was shorter in IWU protocol than in TWU. It appears that, in IWU, the prolonged TUT during eccentric phase and brief episodes of very high activity in the SSC, improved muscle action (explosive strength) for a longer period. Applying strength training with accentuated eccentric actions is crucial to increase players’ ability to stop, cut, and go^2^. Moreover, adding a perceptual-cognitive demand, as in IWU, may add cognitive stimuli, especially useful for young athletes [30].

With regards to 40 m, there were no differences between the two WU modalities. Thus, it appears that this complex motor-performance is less likely to be affected, either positively or negatively, by both the WUs.

Although the dynamic-type loading used in this study facilitated the function of the neuromuscular system, no tests on neuromuscular activation were performed in our investigation.

However, it was hypothesized that explosive strength improvements were caused by longer TUT of the lower limb muscles, we did not assess it in this study.

### Practical Implications

These results may have useful perspectives and practical applications on WU and training organization in youth soccer. The IWU allows the athlete to transfer the accentuated eccentric load effects to the real sport performance. The task-specific condition, in which IWU was performed, guaranteed significant and longer-lasting improvements in CODs. Moreover, IWU included perceptual stimuli which might arouse the decision-making combined with strength improvements. It is appropriate to improve agility performance in young players.

These findings suggest that it is important to specifically apply accentuated eccentric load on sport movements, soliciting intermuscular coordination patterns in order to maximize the transfer in the successive soccer performance.

Considering that IWU outcomes were the same or better than those of TWU, this modality could be recommended for young athletes. The use of this device for coaches may be an affordable training choice, reducing the training times, as a variation of training means and methodologies. In young soccer players’ WU, the flywheel application might be realized as a part of a circuit training, using only one or two devices for all the team.

## 5. Conclusions

The findings of this study showed that isoinertial eccentric overload warm-up produced immediate performance improvements. Flywheel application may have had a role of PAP in young soccer players’ WU, due to a prior high intensity and prolonged muscle activation, which have improved strength and performance.

The most interesting result of this study was the IWU longer-lasting effect on the analyzed variables. Greater TUT, carried out during the accentuated eccentric load, may have led to superior strength gains, as long as the training load did not cause excessive fatigue.

Moreover, the isoinertial device may be applied directly on specific sport conditions, overloading multidirectional soccer movements (forward, backward, and lateral), in different joint angles.

### Limitations

The findings of this study should be viewed in the context of the following limitations:

- Although the dynamic-type loading used in this study facilitated the function of the neuromuscular system, no tests on neuromuscular activation were performed in our investigation. However, it was hypothesized that explosive strength improvements were caused by longer TUT of the lower limb muscles; we did not assess it in this study.
- The eccentric overload amount was not adjusted on an individual-player basis.
- Focusing the intervention on young soccer players, it is better not to generalize the results to a higher level of expertise athletes.

## Figures and Tables

**Table 1:** Report of the full statistics for the main effects of warm-up, time, and the warm-up by time.

Variables	Effects	*DoF*	*F*	*p*	*1–β*	*µ^2^_p_*
**40 m sprint Time**						
	*Warm-Up*	*1.11*	*1.16*	*0.23*	*0.21*	*0.12*
	*Time*	*3.33*	*1.20*	*0.32*	*0.29*	*0.09*
	Warm-Up by Time *	*3.33*	*3.36*	*0.03*	*0.70*	*0.23*
**Illinois Agility Test**						
	*Warm-Up*	*1.11*	*3.24*	*0.09*	*0.37*	*0.22*
	Time *	*3.33*	*53.72*	*<0.001*	*0.98*	*0.83*
	Warm-Up by Time *	*3.33*	*6.94*	*<0.001*	*0.96*	*0.38*
**Squat Jump flight time**						
	Warm-Up *	*1.11*	*12.37*	*0.004*	*0.89*	*0.52*
	Time *	*3.33*	*15.38*	*0.002*	*0.99*	*0.58*
	Warm-Up by Time *	*3.33*	*4.41*	*0.01*	*0.83*	*0.28*
**Countermovement Jump flight time**						
	Warm-Up *	*1.11*	*17.44*	*<0.001*	*0.96*	*0.61*
	Time *	*333*	*13.10*	*<0.001*	*0.99*	*0.54*
	Warm-Up by Time *	*3.33*	*3.61*	*0.02*	*0.74*	*0.24*
**Drop Jump flight time**						
	Warm-Up *	*1.11*	*8.88*	*0.012*	*0.77*	*0.44*
	Time*	*3.33*	*33.72*	*<0.001*	*0.99*	*0.75*
	Warm-Up by Time *	*3.33*	*5.44*	*<0.003*	*0.90*	*0.33*
**Drop Jump contact time**						
	Warm-Up *	*1.11*	*21.45*	*<0.001*	*0.98*	*0.66*
	*Time*	*3.33*	*<1*	*0.99*	*0.05*	*0.02*
	Warm-Up by Time *	*3.33*	*3.45*	*0.02*	*0.72*	*0.23*
**7R-HOP flight time**						
	Warm-Up *	*1.11*	*29.07*	*<0.001*	*0.99*	*0.72*
	Time *	*3.33*	*22.26*	*<0.001*	*0.99*	*0.66*
	Warm-Up by Time *	*3.33*	*8.43*	*0.01*	*0.98*	*0.43*
**7R-HOP contact time**						
	*Warm-Up*	*1.11*	*<1*	*0.88*	*0.05*	*0.01*
	*Time*	*3.33*	*<1*	*0.49*	*0.20*	*0.06*
	*Warm-Up by Time*	*3.33*	*<1*	*0.99*	*0.05*	*0.01*

* Denotes significant effects. DoF = degrees of freedom; *1–β* = achieved power; *µ^2^_p_* = partial eta squared; *F* = Fisher’s F-value; *p* = *p*-value.
